# Nb_1.30_Cr_0.70_S_5_: a layered ternary mixed-metal sulfide

**DOI:** 10.1107/S1600536808041585

**Published:** 2008-12-13

**Authors:** Hoseop Yun, Gangbeom Kim

**Affiliations:** a Division of Energy Systems Research and Department of Chemistry, Ajou University, Suwon 443-749, Republic of Korea

## Abstract

The new layered ternary sulfide, Nb_1.30_Cr_0.70_S_5_, niobium chromium penta­sulfide, is isostructural with the solid solution Nb_1+*x*_V_1−*x*_S_5_ and belongs to the FeNb_3_Se_10_ structure type. Each layer is composed of two unique chains of face-sharing [NbS_8_] bicapped trigonal prisms (*m* symmetry) and edge-sharing [*M*S_6_] (*M*= Nb, Cr) octa­hedra (*m* symmetry). One of the two metal sites is occupied by statistically disordered Nb and Cr atoms, with 0.3 and 0.7 occupancy, respectively. The chains are connected along the *c* axis, forming two-dimensional layers, which then stack on top of each other to complete the three dimensional structure. As a result, an undulating van der Waals gap is found between the layers.

## Related literature

The title compound is isostructural with FeNb_3_Se_10_ (Meerschaut *et al.*, 1981 ▶), Cr_1.70_Nb_2.30_Se_10_ (Mori *et al.*, 1984 ▶) and Nb_1+*x*_V_1−*x*_S_5_ (Yun *et al.*, 2003 ▶). For the structure of a related niobium sulfide, see: Rijnsdorp & Jellinek (1978 ▶). For ionic radii, see: Shannon (1976 ▶). For related literature and background, see: Kim & Yun (2002 ▶); Gelato & Parthé (1987 ▶).

## Experimental

### 

#### Crystal data

Cr_0.70_Nb_1.30_S_5_
*M* *_r_* = 317.48
Monoclinic, 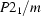

*a* = 8.7938 (14) Å
*b* = 3.3638 (5) Å
*c* = 9.9565 (16) Å
β = 115.193 (3)°
*V* = 266.50 (7) Å^3^
*Z* = 2
Mo *K*α radiation
μ = 5.98 mm^−1^
*T* = 150 (1) K
0.45 × 0.11 × 0.10 mm

#### Data collection

Rigaku R-AXIS RAPID diffractometer
Absorption correction: numerical (*NUMABS*; Higashi, 2000 ▶) *T*_min_ = 0.442, *T*_max_ = 0.543
1979 measured reflections
889 independent reflections
795 reflections with *I* > 2σ(*I*)
*R*_int_ = 0.030

#### Refinement

*R*[*F* ^2^ > 2σ(*F* ^2^)] = 0.048
*wR*(*F* ^2^) = 0.079
*S* = 1.18
889 reflections
44 parameters
Δρ_max_ = 1.21 e Å^−3^
Δρ_min_ = −1.49 e Å^−3^

### 

Data collection: *RAPID-AUTO* (Rigaku, 2006 ▶); cell refinement: *RAPID-AUTO*; data reduction: *RAPID-AUTO*; program(s) used to solve structure: *SHELXS97* (Sheldrick, 2008 ▶); program(s) used to refine structure: *SHELXL97* (Sheldrick, 2008 ▶); molecular graphics: locally modified version of *ORTEP* (Johnson, 1965 ▶); software used to prepare material for publication: *WinGX* (Farrugia, 1999 ▶).

## Supplementary Material

Crystal structure: contains datablocks global, I. DOI: 10.1107/S1600536808041585/wm2207sup1.cif

Structure factors: contains datablocks I. DOI: 10.1107/S1600536808041585/wm2207Isup2.hkl

Additional supplementary materials:  crystallographic information; 3D view; checkCIF report

## Figures and Tables

**Table:** 

Nb1—S1^i^	2.5067 (14)
Nb1—S3^i^	2.5265 (14)
Nb1—S4^ii^	2.5266 (14)
Nb1—S5	2.5814 (17)
Nb1—S1^iii^	2.6065 (17)
*M*—S2	2.328 (2)
*M*—S5^i^	2.4159 (15)
*M*—S2^iv^	2.4190 (15)
*M*—S1	2.4980 (19)
S3—S4	2.047 (2)

**Table:** 

S1^i^—Nb1—S1^ii^	84.28 (6)
S1^i^—Nb1—S3^i^	89.97 (4)
S1^ii^—Nb1—S3^i^	153.23 (6)
S3^i^—Nb1—S3^ii^	83.47 (6)
S1^i^—Nb1—S4^ii^	158.17 (6)
S1^ii^—Nb1—S4^ii^	92.00 (4)
S3^i^—Nb1—S4^ii^	102.39 (5)
S3^ii^—Nb1—S4^ii^	47.79 (5)
S1^i^—Nb1—S5	78.25 (5)
S3^i^—Nb1—S5	126.13 (4)
S4^ii^—Nb1—S5	79.92 (5)
S1^i^—Nb1—S1^iii^	73.94 (5)
S3^i^—Nb1—S1^iii^	79.34 (5)
S4^ii^—Nb1—S1^iii^	125.68 (4)
S2—*M*—S5^i^	94.92 (6)
S5^i^—*M*—S5^ii^	88.24 (7)
S2—*M*—S2^iv^	95.59 (6)
S5^i^—*M*—S2^iv^	169.50 (7)
S5^ii^—*M*—S2^iv^	90.87 (4)
S2^iv^—*M*—S2^v^	88.10 (7)
S2—*M*—S1	175.11 (7)
S5^i^—*M*—S1	81.60 (6)
S2^iv^—*M*—S1	87.92 (5)

Symmetry codes: (i) 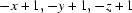; (ii) 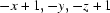; (iii) 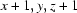; (iv) 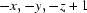; (v) 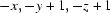.

**Fig. 1.**
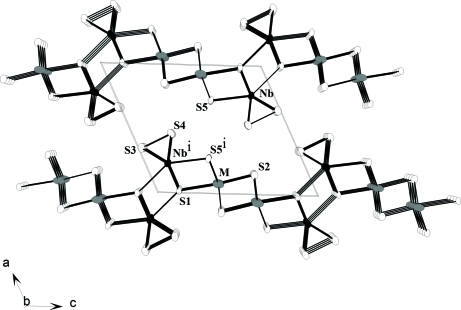
A perspective view of Nb1.30Cr0.70S5 down the b axis showing the stacking of the layers. The M site is occupied by statistically disordered Nb(30%) and Cr(70%) atoms. Filled, gray, and open circles represent Nb, M(Nb or Cr), and S atoms, repectively. Displacement ellipsoids are drawn at the 90% probability level. [Symmetry code: (i) 1 - x, 1/2 + y, 1 - z.]

**Fig. 2.**
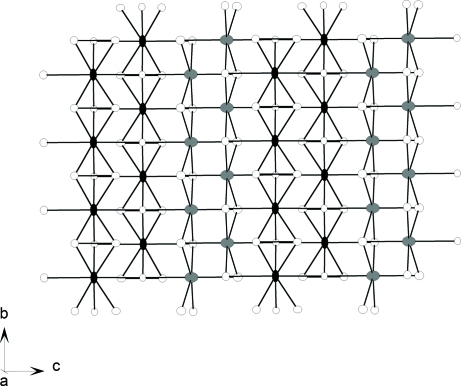
View of Nb1.30Cr0.70S5 along the a axis, showing the individual layer and the coordination around the metal atoms.

## supplementary crystallographic information

### Comment

The title compound is isostructural with FeNb_3_Se_10_ (Meerschaut *et al.*, 1981), Cr_1.70_Nb_2.30_Se_10_ (Mori *et al.*, 1984), and the solid solution Nb_1+_*_x_*V_1-_*_x_*S_5_ (Yun *et al.*, 2003).

A view down the *b*-axis of Nb_1.30_Cr_0.70_S_5_ shows the layered nature of the structure (Figure 1). Figure 2 shows that an individual layer is composed of two unique chains of face-sharing [NbS_8_]  bicapped trigonal prisms and edge-sharing [*M*S_6_] (*M*= Nb, Cr) octahedra. The Nb atom is surrounded by six S atoms in a distorted trigonal-prismatic fashion. Atoms S1, S3, and S4 form an isosceles triangle, the S3—S4 distance (2.047 (2) Å) being much shorter than the other two (> 3.0 A). This short S3—S4 separation is typical of (S—S)^2-^ pairs (Kim & Yun, 2002). The Nb atoms are further coordinated by two additional S atoms that cap two of the rectangular faces of the trigonal prism. The Nb—S distances, ranging from 2.507 (1) to 2.607 (2) Å, are in agreement with the usual Nb—S distances found in niobium sulfides such as NbS_3_ (Rijnsdorp & Jellinek, 1978). Longer Nb—S distances are observed for the capping S5 atoms. The Nb-centered bicapped trigonal prisms share their triangular faces to form a one-dimensional chain along the direction of the *b*-axis. Two of these chains are linked together by sharing two S1 atoms to form a double bicapped trigonal prismatic chain, [Nb_2_S_8_].

The *M*2 site, occupied by 30% of Nb and 70% of Cr, is surrounded by six S atoms in a distorted octahedral fashion. These octahedra then share their edges through atoms S2 and S5 to form a one-dimensional chain. Again, two octahedral chains are bound by sharing two S2 atoms and thus form a double chain, [*M*_2_S_6_]. In spite of the partial occupation of Nb, the M—S distances are in good agreement with that calculated from their ionic radii (2.455 Å, Shannon, 1976). This structural unit allows significant interchain zigzag metal–metal interactions, and an intermediate *M*—*M* separation (3.190 (2) Å) is found. The intrachain *M*—*M* distance, which is significantly longer than the interchain *M*—*M* distance, is the same as the repeating unit along the *b*-axis (3.3638 (5) Å).

These double Nb and *M*-centered chains are condensed together through atoms S1 and S5, and a quadruple chain of composition [Nb_2_*M*_2_S_12_] is completed. Finally, these chains are connected along the *c* axis to form a two-dimensional layer, ^2^_∞_[Nb*M*S_5_]. These layers then stack on top of each other to form the three-dimensional structure with an undulating van der Waals gap, as shown in Figure 1. There is no bonding interaction, only van der Waals forces, between these layers.

### Experimental

The title compound, Nb_1.30_Cr_0.70_S_5_ was obtained from a reaction of Nb, Cr, and S in an elemental ratio of 1:1:5 in the presence of LiCl as flux. The mass ratio of reactants and flux was 1:3. The starting materials were placed in a fused-silica tube. The tube was evacuated to 0.133 Pa, sealed, and heated to 973 K at a rate of 80 K/hr, where it was kept for 7 days. The tube was cooled at a rate of 4 K/hr to 373 K and the furnace was shut off. Air- and water-stable black needle-shaped crystals were isolated after the flux was removed with water. Qualitative analysis of the crystals with an EDAX-equipped scanning electron microscope indicated the presence of Nb, Cr, and S. No other element was detected.

### Refinement

With the stoichiometric NbCrS_5_ model, the displacement parameters for the *M*2 site are significantly smaller than those of the other atoms, which suggests that this site may be shared by Cr and Nb atoms. The positional and anisotropic displacement parameters (ADPs) of Nb and Cr in this site are equated by constraints. The result of the refinement was improved significantly by introducing the disordered model, and the displacement parameters became more plausible. The best fit was found when the *M*2 site was refined with site occupancy factors (s.o.f.) of 30% for Nb and 70% for Cr. With the composition established, the s.o.f.'s were fixed and the data were finally corrected for absorption with the use of the numerical method. The structure was standardized by means of the program *STRUCTURE TIDY* (Gelato & Parthé, 1987).

### Crystal data

**Table:**

Cr_0.70_Nb_1.30_S_5_	*F*(000) = 300
*M**_r_* = 317.48	*D*_x_ = 3.956 Mg m^−^^3^
Monoclinic, *P*2_1_/*m*	Mo *K*α radiation, λ = 0.71073 Å
Hall symbol: -P 2yb	Cell parameters from 3442 reflections
*a* = 8.7938 (14) Å	θ = 3.2–27.5°
*b* = 3.3638 (5) Å	µ = 5.98 mm^−^^1^
*c* = 9.9565 (16) Å	*T* = 150 K
β = 115.193 (3)°	Needle, black
*V* = 266.50 (7) Å^3^	0.45 × 0.11 × 0.10 mm
*Z* = 2	

### Data collection

**Table:**

Rigaku R-AXIS RAPID diffractometer	795 reflections with *I* > 2σ(*I*)
graphite	*R*_int_ = 0.030
ω scans	θ_max_ = 30.0°, θ_min_ = 2.3°
Absorption correction: numerical (*NUMABS*; Higashi, 2000)	*h* = −12→9
*T*_min_ = 0.442, *T*_max_ = 0.543	*k* = −4→3
1979 measured reflections	*l* = −13→14
889 independent reflections	

### Refinement

**Table:**

Refinement on *F*^2^	0 restraints
Least-squares matrix: full	Primary atom site location: structure-invariant direct methods
*R*[*F*^2^ > 2σ(*F*^2^)] = 0.048	Secondary atom site location: difference Fourier map
*wR*(*F*^2^) = 0.079	*w* = 1/[σ^2^(*F*_o_^2^) + (0.0234*P*)^2^ + 1.143*P*] where *P* = (*F*_o_^2^ + 2*F*_c_^2^)/3
*S* = 1.18	(Δ/σ)_max_ < 0.001
889 reflections	Δρ_max_ = 1.21 e Å^−^^3^
44 parameters	Δρ_min_ = −1.49 e Å^−^^3^

### Special details

**Table:**

Geometry. All s.u.'s (except the s.u. in the dihedral angle between two l.s. planes) are estimated using the full covariance matrix. The cell s.u.'s are taken into account individually in the estimation of s.u.'s in distances, angles and torsion angles; correlations between s.u.'s in cell parameters are only used when they are defined by crystal symmetry. An approximate (isotropic) treatment of cell s.u.'s is used for estimating s.u.'s involving l.s. planes.
Refinement. Refinement of *F*^2^ against ALL reflections. The weighted *R*-factor *wR* and goodness of fit *S* are based on *F*^2^, conventional *R*-factors *R* are based on *F*, with *F* set to zero for negative *F*^2^. The threshold expression of *F*^2^ > 2σ(*F*^2^) is used only for calculating *R*-factors(gt) *etc*. and is not relevant to the choice of reflections for refinement. *R*-factors based on *F*^2^ are statistically about twice as large as those based on *F*, and *R*- factors based on ALL data will be even larger.

### Fractional atomic coordinates and isotropic or equivalent isotropic displacement parameters (Å^2^)

**Table:**

	*x*	*y*	*z*	*U*_iso_*/*U*_eq_	Occ. (<1)
Nb1	0.77552 (8)	0.25	0.86289 (6)	0.00665 (17)	
Nb2	0.06914 (12)	0.25	0.40161 (11)	0.0124 (2)	0.3
Cr2	0.06914 (12)	0.25	0.40161 (11)	0.0124 (2)	0.7
S1	0.0113 (2)	0.25	0.13327 (17)	0.0057 (3)	
S2	0.1474 (2)	0.25	0.65632 (18)	0.0082 (3)	
S3	0.3415 (2)	0.25	0.01649 (18)	0.0080 (3)	
S4	0.4604 (2)	0.25	0.24351 (18)	0.0080 (3)	
S5	0.7293 (2)	0.25	0.58889 (17)	0.0066 (3)	

### Atomic displacement parameters (Å^2^)

**Table:**

	*U*^11^	*U*^22^	*U*^33^	*U*^12^	*U*^13^	*U*^23^
Nb1	0.0057 (3)	0.0112 (4)	0.0035 (3)	0	0.0024 (2)	0
Nb2	0.0076 (4)	0.0097 (5)	0.0169 (5)	0	0.0023 (4)	0
Cr2	0.0076 (4)	0.0097 (5)	0.0169 (5)	0	0.0023 (4)	0
S1	0.0078 (7)	0.0052 (8)	0.0042 (7)	0	0.0027 (6)	0
S2	0.0104 (8)	0.0070 (9)	0.0082 (7)	0	0.0050 (6)	0
S3	0.0121 (8)	0.0049 (9)	0.0073 (7)	0	0.0045 (6)	0
S4	0.0105 (8)	0.0061 (9)	0.0058 (7)	0	0.0020 (6)	0
S5	0.0087 (7)	0.0067 (8)	0.0060 (7)	0	0.0046 (6)	0

### Geometric parameters (Å, °)

**Table:**

Nb1—S1^i^	2.5067 (14)	M—M^iv^	3.1898 (18)
Nb1—S1^ii^	2.5067 (14)	S1—Nb1^i^	2.5067 (14)
Nb1—S3^i^	2.5265 (14)	S1—Nb1^ii^	2.5067 (14)
Nb1—S3^ii^	2.5265 (14)	S1—Nb1^vi^	2.6065 (16)
Nb1—S4^ii^	2.5266 (14)	S2—M^iv^	2.4190 (15)
Nb1—S4^i^	2.5266 (14)	S2—Nb2^v^	2.4190 (15)
Nb1—S5	2.5814 (17)	S3—S4	2.047 (2)
Nb1—S1^iii^	2.6065 (17)	S3—Nb1^i^	2.5265 (14)
M—S2	2.328 (2)	S3—Nb1^ii^	2.5265 (14)
M—S5^i^	2.4159 (15)	S4—Nb1^ii^	2.5266 (14)
M—S5^ii^	2.4159 (15)	S4—Nb1^i^	2.5266 (14)
M—S2^iv^	2.4190 (15)	S5—M^i^	2.4159 (15)
M—S2^v^	2.4190 (15)	S5—Cr2^ii^	2.4159 (15)
M—S1	2.4980 (19)	S5—M^ii^	2.4159 (15)
M—M^v^	3.1898 (18)		
			
S1^i^—Nb1—S1^ii^	84.28 (6)	S2^iv^—M—S2^v^	88.10 (7)
S1^i^—Nb1—S3^i^	89.97 (4)	S2—M—S1	175.11 (7)
S1^ii^—Nb1—S3^i^	153.23 (6)	S5^i^—M—S1	81.60 (6)
S1^i^—Nb1—S3^ii^	153.23 (6)	S5^ii^—M—S1	81.60 (6)
S1^ii^—Nb1—S3^ii^	89.97 (4)	S2^iv^—M—S1	87.92 (5)
S3^i^—Nb1—S3^ii^	83.47 (6)	S2^v^—M—S1	87.92 (5)
S1^i^—Nb1—S4^ii^	158.17 (6)	S2—M—M^v^	49.01 (4)
S1^ii^—Nb1—S4^ii^	92.00 (4)	S5^i^—M—M^v^	94.25 (4)
S3^i^—Nb1—S4^ii^	102.39 (5)	S5^ii^—M—M^v^	143.92 (6)
S3^ii^—Nb1—S4^ii^	47.79 (5)	S2^iv^—M—M^v^	92.63 (6)
S1^i^—Nb1—S4^i^	92.00 (4)	S2^v^—M—M^v^	46.58 (4)
S1^ii^—Nb1—S4^i^	158.17 (6)	S1—M—M^v^	134.40 (5)
S3^i^—Nb1—S4^i^	47.79 (5)	S2—M—M^iv^	49.01 (4)
S3^ii^—Nb1—S4^i^	102.39 (5)	S5^i^—M—M^iv^	143.92 (6)
S4^ii^—Nb1—S4^i^	83.47 (6)	S5^ii^—M—M^iv^	94.25 (4)
S1^i^—Nb1—S5	78.25 (5)	S2^iv^—M—M^iv^	46.58 (4)
S1^ii^—Nb1—S5	78.25 (5)	S2^v^—M—M^iv^	92.63 (6)
S3^i^—Nb1—S5	126.13 (4)	S1—M—M^iv^	134.40 (5)
S3^ii^—Nb1—S5	126.13 (4)	M^v^—M—M^iv^	63.64 (4)
S4^ii^—Nb1—S5	79.92 (5)	M—S1—Nb1^i^	99.97 (5)
S4^i^—Nb1—S5	79.92 (5)	M—S1—Nb1^ii^	99.97 (5)
S1^i^—Nb1—S1^iii^	73.94 (5)	Nb1^i^—S1—Nb1^ii^	84.28 (6)
S1^ii^—Nb1—S1^iii^	73.94 (5)	M—S1—Nb1^vi^	144.58 (8)
S3^i^—Nb1—S1^iii^	79.34 (5)	Nb1^i^—S1—Nb1^vi^	106.06 (5)
S3^ii^—Nb1—S1^iii^	79.34 (5)	Nb1^ii^—S1—Nb1^vi^	106.06 (5)
S4^ii^—Nb1—S1^iii^	125.68 (4)	M—S2—M^iv^	84.41 (6)
S4^i^—Nb1—S1^iii^	125.68 (4)	M—S2—M^v^	84.41 (6)
S5—Nb1—S1^iii^	142.16 (6)	M^iv^—S2—M^v^	88.10 (7)
S2—M—S5^i^	94.92 (6)	S4—S3—Nb1^i^	66.11 (6)
S2—M—S5^ii^	94.92 (6)	S4—S3—Nb1^ii^	66.11 (6)
S5^i^—M—S5^ii^	88.24 (7)	Nb1^i^—S3—Nb1^ii^	83.47 (6)
S2—M—S2^iv^	95.59 (6)	S3—S4—Nb1^ii^	66.10 (6)
S5^i^—M—S2^iv^	169.50 (7)	S3—S4—Nb1^i^	66.10 (6)
S5^ii^—M—S2^iv^	90.87 (4)	Nb1^ii^—S4—Nb1^i^	83.47 (6)
S2—M—S2^v^	95.59 (6)	M^i^—S5—M^ii^	88.24 (7)
S5^i^—M—S2^v^	90.87 (4)	M^i^—S5—Nb1	100.12 (5)
S5^ii^—M—S2^v^	169.50 (7)	M^ii^—S5—Nb1	100.12 (5)

Symmetry codes: (i) −*x*+1, −*y*+1, −*z*+1; (ii) −*x*+1, −*y*, −*z*+1; (iii) *x*+1, *y*, *z*+1; (iv) −*x*, −*y*, −*z*+1; (v) −*x*, −*y*+1, −*z*+1; (vi) *x*−1, *y*, *z*−1.
